# Association of neutrophil-to-lymphocyte ratio and initial facial weakness with electroneurography results in early-stage Bell’s palsy: A retrospective cohort study

**DOI:** 10.1097/MD.0000000000049548

**Published:** 2026-06-26

**Authors:** Seongmin Choi, Myung Chul Yoo

**Affiliations:** a Department of Physical Medicine & Rehabilitation, College of Medicine, Kyung Hee University, Seoul, Republic of Korea; b Department of Medicine, Graduate School, Kyung Hee University, Seoul, Republic of Korea.

**Keywords:** Bell’s palsy, electroneurography, neutrophil-to-lymphocyte ratio

## Abstract

This study aimed to investigate the clinical factors, including neutrophil-to-lymphocyte ratio (NLR) and the initial House-Brackmann (H-B) grade, associated with electroneurography (ENoG) in patients with early-stage Bell’s palsy. This retrospective cohort study evaluated 97 adults (≥18 years) with early-stage Bell’s palsy diagnosed at our university hospital between January 2014 and September 2021. The medical records of these patients, including the NLR and the initial H-B grade, were reviewed. The degree of axonal loss was calculated using the compound muscle action potential (CMAP) amplitude ratio measured by ENoG. Patients were classified into 2 groups based on the degree of axonal loss: poor and good results, defined as >90% loss of amplitude (ENoG results < 10%) and ≤90% loss of amplitude (ENoG results ≥ 10%), respectively. Multivariable analysis revealed that an initial H-B grade II–III (odds ratio [OR], 4.79; 95% confidence interval [CI], 1.39–16.46) and an NLR < 1.92 (OR, 5.54; 95% CI, 1.85–16.53) were associated with good ENoG results. In our construction of a classification and regression tree (CART) model that considered various variables to predict the degree of axonal loss, the first partitioning node was initial H-B grade II, and CART analysis with minimal classification error found that an NLR of 1.61 was the second partitioning predictor. The model showed a sensitivity, specificity, and an accuracy of 87.5%, 80%, and 85.6%, respectively. By measuring the NLR at the time of Bell’s palsy onset and initial facial weakness evaluated by H-B grade, it may be possible to predict subsequent axonal loss calculated by ENoG, which is performed 10 to 14 days after symptom onset.

## 1. Introduction

Approximately half of all patients with facial nerve palsy experience Bell’s palsy, a condition characterized by acute unilateral peripheral facial palsy.^[1]^ The annual incidence rate ranges from 13 to 34 per 1,00,000 individuals.^[2]^ The pathophysiology of Bell’s palsy follows inflammation and cytotoxic edema of the facial nerve fibers following viral infection, with lymphocyte infiltration being responsible for demyelination and axonal degeneration.^[3,4]^ The reactivation of latent herpes simplex virus type 1 or varicella-zoster virus within the geniculate ganglia is the major cause of Bell’s palsy.^[5]^ Though the definitive mechanism remains unclear, Bell’s palsy is also thought to be due to microcirculation disorders in the small arteries that supply blood to the facial nerve, ischemic neuropathy, and infectious and immunological causes.^[6]^

Several studies have investigated the electrophysiological tests predicting the prognosis of patients with Bell’s palsy.^[7,8]^ Clinical assays, including stapedius reflex tests, electromyography (EMG), nerve excitability tests, electroneurography (ENoG), and blink reflex tests, may predict the prognosis of patients with early stages of Bell’s palsy.^[7]^ ENoG is crucial for electrophysiological assessment, as well as for investigating the risk of poor outcomes during the early stages of acute facial palsy. ENoG tests measure the severity of facial nerve injury as the percentage of the maximal amplitude on the affected side divided by the maximal amplitude on the unaffected side. ENoG can quantify facial nerve function by recording motor unit and/or compound muscle action potentials (CMAP). Estimating the amount of nerve degeneration can be enabled by comparing the peak-to-peak amplitude of the CMAPs on the affected and unaffected sides. Patients with >90% degeneration on the affected side within 14 days of facial paralysis onset are considered to have poor outcomes.^[8]^

White blood cell (WBC) counts and WBC subtype counts are useful inflammatory biomarkers in clinical practice. The neutrophil-to-lymphocyte ratio (NLR) is a readily available, inexpensive, relatively noninvasive, and novel potential laboratory marker that is used to measure systemic inflammation.^[9,10]^ The NLR has been utilized to diagnose and predict the prognosis of patients with Bell’s palsy. The NLR has been reported to be elevated in patients with Bell’s palsy and could therefore be an early predictor of prognosis in patients with idiopathic peripheral facial palsy.^[11,12]^

Although ENoG tests can objectively determine the initial degree of facial nerve injury as axonal loss, the correlation between the severity of facial nerve injury determined by ENoG, initial facial weakness, and NLR in patients with early-stage Bell’s palsy has not yet been determined. We hypothesized that the NLR might be indicative of the severity of facial nerve injury, and also supposed that the initial facial weakness evaluated on the House-Brackmann (H-B) grade might be related to the results of the ENoG test. If the NLR and initial H-B grade were associated with the ENoG test, it may be possible to predict subsequent axonal loss earlier. Therefore, the present study investigated the relationship between NLR, initial facial weakness presented as H-B grade, and severity of facial nerve injury, as determined by comparing the amplitude of CMAP on the affected and unaffected sides.

## 2. Materials and methods

### 2.1. Study population and design

**Figure 1.**
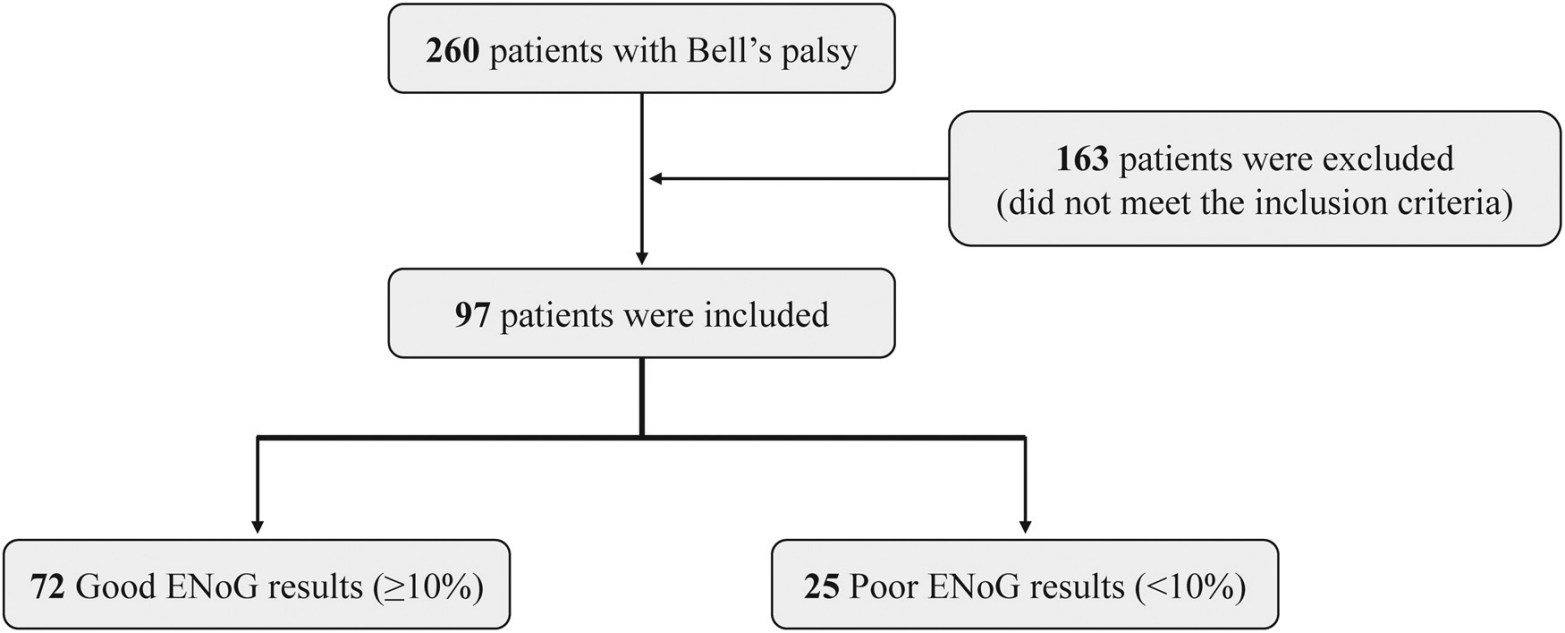
Study flowchart. ENoG = electroneurography.

This retrospective study included patients aged ≥18 years who were diagnosed with Bell’s palsy at our university hospital between January 2014 and September 2021. The inclusion criteria were: patients with acute, unilateral, isolated peripheral facial palsy. We reviewed the medical records of 260 patients admitted to our university hospital for the management of acute facial palsy during this period. Of these 260 patients, 163 were excluded because they had Ramsay-Hunt syndrome, otitis media, facial palsy after head and/or neck surgery, a previous history of oral steroid therapy, recurrent Bell’s palsy, unclear timing of blood tests, and/or acute systemic infection (WBC > 11,000/µL). No patients with initial H-B grade VI were included in the final analysis. Finally, 97 patients were included in this study (Fig. 1). Demographic and clinical characteristics, including age, sex, body mass index (BMI), presence of comorbidities such as hypertension and diabetes mellitus, HbA1c, and electrophysiological test results, were obtained from patient records (Table 1). In addition, blood tests were performed on all subjects at admission, and complete blood counts (CBC) were performed to assess white blood cell (WBC), neutrophil, and lymphocyte counts. The NLR was calculated as the ratio of neutrophil count to lymphocyte count. The initial severity of facial weakness was also evaluated using the H-B scale and dichotomized as H-B grades II–III and IV–V.

**Table 1:** Baseline demographic and clinical characteristics in patients with good and poor ENoG results.

Variable	Good ENoG results (n = 72)	Poor ENoG results (n = 25)	*P*-value
Age (yr)			
<40	15 (20.8%)	2 (8%)	.22
≥40	57 (79.2%)	23 (92%)	
Sex			
Male	40 (55.6%)	13 (52%)	.76
Female	32 (44.4%)	12 (48%)	
BMI (kg/m^2^)	25.0 ± 3.7	25.3 ± 3.0	.34
Underlying disease			
HTN	31 (43.1%)	13 (52%)	.44
DM	23 (31.9%)	11 (44%)	.28
Initial H-B-grade			
II–III	64 (88.9%)	16 (64%)	.01*
IV–V	8 (11.1%)	9 (36%)	
Blood test			
HbA1c (%)	6.1 ± 0.9	6.8 ± 1.8	.20
Neutrophils (%)	55.5 ± 9.5	63.3 ± 9.2	<.01*
Lymphocytes (%)	33.9 ± 8.1	27.0 ± 7.1	<.01*
WBC (10^3^/µL)	6.89 ± 1.60	7.24 ± 1.56	.34
Platelets (10^3^/µL)	233.3 ± 48.9	241.4 ± 65.2	.44
NLR	1.83 ± 0.90	2.62 ± 1.14	<.01*

Values are presented as number (%) or as mean ± standard deviation.

BMI = body mass index, DM = diabetes mellitus, ENoG = electroneurography, H-B grade = House-Brackmann grade, HbA1c = glycated hemoglobin, HTN = hypertension, NLR = neutrophil-to-lymphocyte ratio, WBC = white blood cell.

* *P* < .05.

### 2.2. Electrodiagnostic evaluation

The severity of facial nerve injury was evaluated electrophysiologically, including with ENoG tests. All subjects underwent ENoG examination 10 to 14 days after facial palsy. Nerve conduction studies (NCS) were performed using electrodes 10 mm in diameter (MT-R20, HUREV LLC., Miami), with recording and stimulation performed using standard protocols.^[13]^ The potentials were recorded from the frontalis, orbicularis oculi, nasalis, and orbicularis oris muscles on each side. By comparing the peak-to-peak amplitudes of CMAPs on the affected and unaffected sides, the magnitude of nerve degeneration was estimated. Because axonal loss >90% is considered a critical indicator of poor outcome, the cutoff value was set at >90% axonal loss, as calculated by the CMAP amplitude ratio. Patients were therefore classified into 2 groups based on the degree of axonal loss, with a poor outcome defined as >90% loss of amplitude (ENoG results < 10%) and a good outcome defined as ≤90% loss of amplitude (ENoG results ≥ 10%; Fig. 1).^[14]^

Decision trees are a type of classification algorithm that is relatively easy to interpret and apply in clinical practice. Classification and regression tree (CART) analysis is a tree-building method that uses binary recursive partitioning.^[15]^ The CART analysis selects the best predictive variable for splitting the data into 2 daughter nodes with maximal purity. Thus, the CART model can be easily applied in clinical settings to assist in clinical decision-making. Therefore, a decision tree using CART analysis was added to determine the optimal combination of variables with good ENoG results. The protocol of this retrospective study was approved by the Institutional Review Board of our university hospital (KHUH 2021-12-034), which waived the requirement for written informed consent because of the retrospective nature of the study.

### 2.3. Statistical analysis

Categorical variables are reported as numbers (%) and compared using Pearson chi-square test. Continuous variables are reported as mean ± standard deviation and compared using the Mann–Whitney *U* test or Student *t* test. Odds ratios (ORs) and 95% confidence intervals (CIs) were calculated using multivariate logistic regression. Receiver operating characteristic (ROC) curve analysis was used to determine the most appropriate cutoff values. The diagnostic performance of the decision trees using the CART model was analyzed, and the sensitivity, specificity, and accuracy of the trees were calculated. CART analyses were performed using R statistical software (version 4.0.0: The R Project for Statistical Computing, Vienna, Austria). Other statistical analyses were performed using the Statistical Package for Social Sciences (version 25.0; SPSS Inc., Chicago), with *P* < .05 defined as statistically significant.

## 3. Results

Of the 260 patients with facial palsy initially enrolled, 163 were excluded because they did not meet the inclusion criteria. The remaining 97 patients with Bell’s palsy comprised 53 (54.6%) men and 44 (45.4%) women with a mean age of 56.48 ± 16.65 years. Of the 97 patients, 44 (45.4%) had hypertension and 34 (35.1%) had diabetes mellitus. Evaluation of the H-B grade showed that 80 patients (82.5%) had initial H-B grades II–III, while 17 (17.5%) had initial H-B grades IV–V.

Table 1 shows the distribution of demographic and clinical variables and their associations with ENoG results. Compared with patients who had poor ENoG results (<10%), those who had good ENoG results (≥10%) had a lower initial H-B grade (II–III, 88.9%; *P* = .01), a lower mean (±SD) neutrophil percentage (55.5 ± 9.5; *P* < .001), a higher lymphocyte percentage (33.9 ± 8.1; *P* < .001), and a lower NLR (1.83 ± 0.90; *P* < .001). However, other factors, including age, sex, BMI, hypertension, and diabetes mellitus, did not differ significantly between the 2 groups.

**Figure 2.**
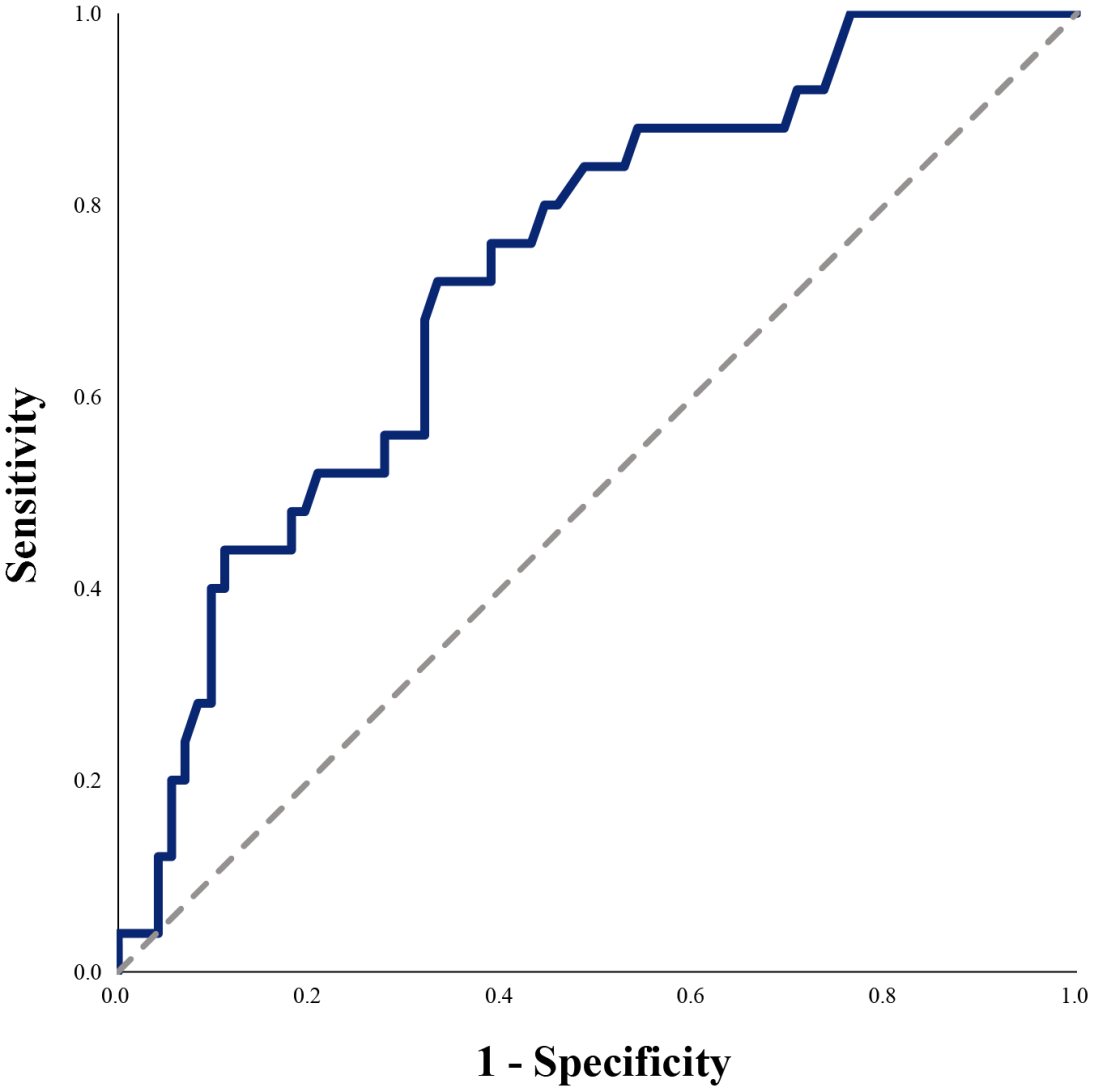
Receiver operating characteristic (ROC) curve analysis of the neutrophil-to-lymphocyte ratio (NLR) for discriminating between good and poor ENoG results. The optimal cutoff value was 1.92. An NLR < 1.92 predicted good ENoG results with a sensitivity of 66.7% and specificity of 72.0%. The area under the curve was 0.731 (95% CI, 0.62–0.84). CI = confidence interval, ENoG = electroneurography, NLR = neutrophil-to-lymphocyte ratio, ROC = receiver operating characteristic.

**Figure 3.**
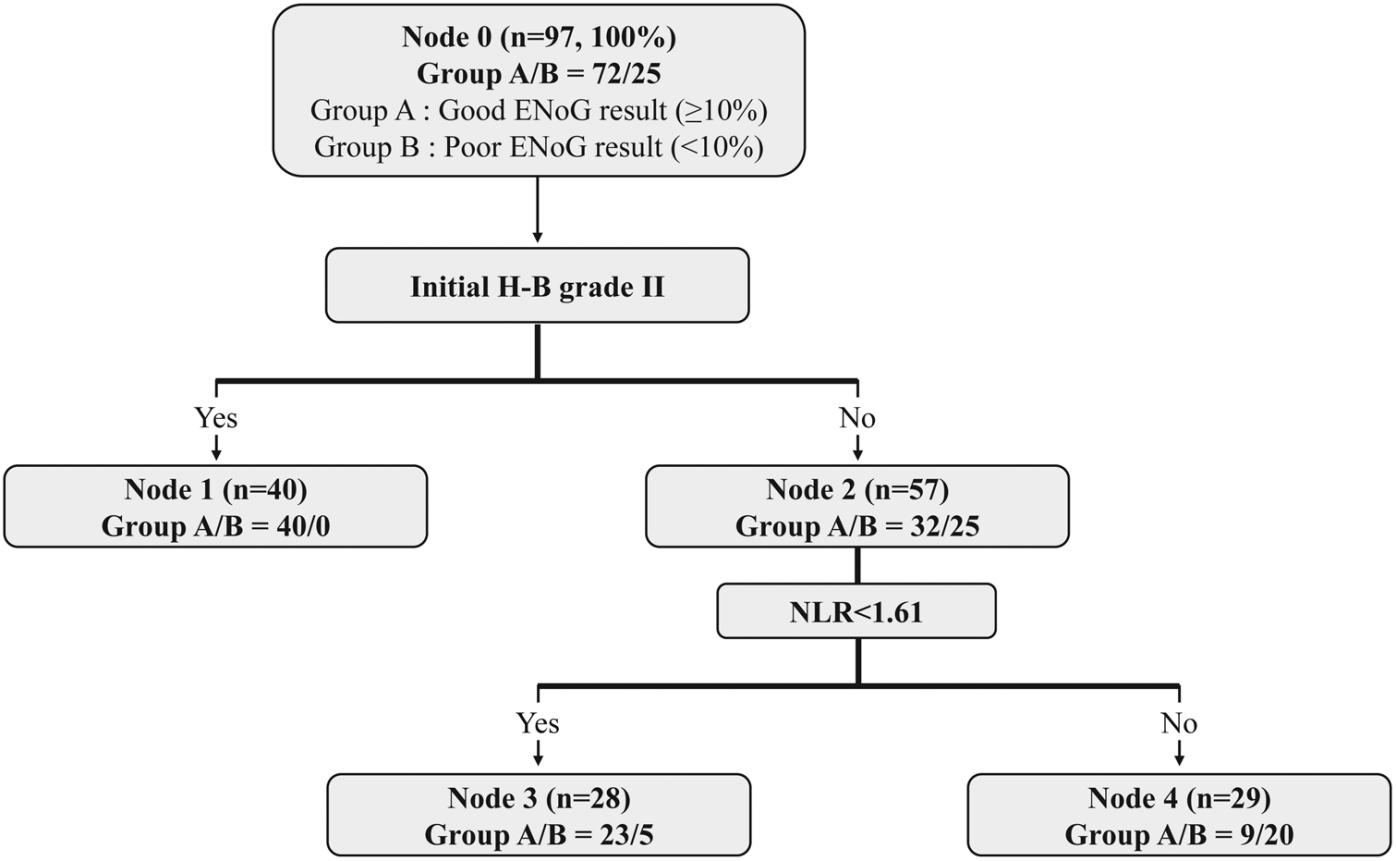
Classification and regression tree (CART) results. The decision-making tree was based on 2 nodes: initial H-B grade II and NLR < 1.61. CART = classification and regression tree, ENoG = electroneurography, H-B = House-Brackmann, NLR = neutrophil-to-lymphocyte ratio.

ROC analysis showed that an NLR < 1.92 was the optimal cutoff value for predicting good ENoG results, with a sensitivity of 66.7% and specificity of 72.0% (Fig. 2). Multivariable logistic regression analysis, including age, sex, hypertension, diabetes mellitus, initial H-B grade, and NLR, demonstrated that an initial H-B grade of II–III (OR, 4.79; 95% CI, 1.39–16.46) and an NLR < 1.92 (OR, 5.54; 95% CI, 1.85–16.53) were significantly associated with good ENoG results (Table 2). A CART decision tree model identified that the initial H-B grade II was the first partitioning node, with none of the patients with initial H-B grade II having poor ENoG results (<10%; Fig. 3). Of the 57 patients with initial H-B grades III–V, 32 (56.1%) had good ENoG results (≥10%), and 25 (43.9%) had poor ENoG results (<10%). When applied to predict good ENoG results, the CART model showed a sensitivity of 87.5%, specificity of 80.0%, and accuracy of 85.6%. Table 3 shows the diagnostic performance of each parameter, including initial H-B grade and NLR, for predicting good ENoG results. The CART model, which incorporated initial H-B grade and NLR, showed the highest overall accuracy for predicting good ENoG results.

**Table 2:** Multivariable logistic regression analysis of factors predicting the probability of good ENoG results in patients with Bell’s palsy.

Variable		Predicting the probability of good ENoG results			
		OR	95% CI		*P*-value
Age (yr)	<40	3.03	0.64	14.30	.17
	≥40	1.00			
Sex	Male	1.02	0.36	2.91	.97
	Female	1.00			
HTN	No	0.68	0.22	2.11	.50
	Yes	1.00			
DM	No	1.12	0.36	3.51	.84
	Yes	1.00			
Initial H-B grade	II–III	4.79	1.39	16.46	.01*
	IV–V	1.00			
NLR	<1.92	5.54	1.85	16.53	<.01*
	≥1.92	1.00			

Data are presented as odds ratios and 95% confidence intervals.

Multivariable logistic regression analysis included age, sex, HTN, DM, initial H-B grade, and NLR as covariates.

H-B grade = House-Brackmann grade, CI = confidence interval, DM = diabetes mellitus, ENoG = electroneurography, HTN = hypertension, NLR = neutrophil-to-lymphocyte ratio, OR = odds ratio.

* *P* < .05.

**Table 3:** Validation: sensitivity, specificity and accuracy of each parameter in predicting good ENoG results.

Variable		Sensitivity (%)	Specificity (%)	Accuracy (%)	*P*-value
Initial H-B grade	II–III	88.89	36.00	75.26	.01*
	IV–V				
NLR	<1.92	66.67	72.00	68.04	<.01*
	≥1.92				
CART	H-B grade II or H-B grade > II & NLR < 1.61	87.50	80.00	85.57	<.01*
	H-B grade > II & NLR ≥ 1.61				

CART = classification and regression tree, H-B grade = House-Brackmann grade, NLR = neutrophil-to-lymphocyte ratio.

* *P* < .05.

## 4. Discussion

This study demonstrated that NLR was independently associated with ENoG-defined axonal loss in patients with early-stage Bell’s palsy. In addition, good ENoG results were associated with a lower initial H-B grade. The CART model incorporating initial H-B grade and NLR predicted good ENoG results with a sensitivity of 87.5%, specificity of 80.0%, and accuracy of 85.6%.

To the best of our knowledge, the correlation between the severity of facial nerve injury, as determined by ENoG, and NLR in patients with Bell’s palsy has not been determined. In the present study, axonal loss in the facial nerves was determined using ENoG measurements of CMAP amplitude and compared with NLR in patients with early Bell’s palsy. Multivariable logistic regression analysis showed that NLR < 1.92 was independently associated with good ENoG results. Conversely, higher NLR values may reflect a greater systemic inflammatory burden, which could be associated with more severe facial nerve edema, demyelination, and axonal degeneration. These results are consistent with previous studies that inflammation may play important roles in Bell’s palsy pathogenesis.^[16–19]^ Liston et al reported histological changes in the facial nerve, in which myelin sheaths were destroyed by infiltration of round, small inflammatory cells.^[20]^ Additionally, the concentration of cytokines, including tumor necrosis factor-alpha (TNF-α), interleukin-1 (IL-1), and interleukin-6 (IL-6) was increased in patients with Bell’s palsy compared to control groups.^[16]^ In addition, the NLR, an indicator of disease severity in inflammatory disorders,^[21–23]^ was reported to be significantly higher in patients with Bell’s palsy than in control individuals, and it was higher in patients with Bell’s palsy who show contrast enhancement of the facial nerve on MRI.^[24]^ Collectively, these findings suggest that inflammatory processes may contribute to facial nerve edema, demyelination, and subsequent axonal degeneration within the narrow facial canal.^[17]^ Because NLR reflects systemic inflammatory burden,^[25]^ elevated NLR levels may indicate a more pronounced inflammatory response affecting the facial nerve. This may explain why patients with higher NLR values demonstrated greater axonal loss, as quantified by ENoG in the present study.

We also found that a lower initial H-B grade was significantly associated with good ENoG results, in agreement with studies showing that the severity of facial weakness at presentation, as determined by the H-B grade, was the main risk factor for incomplete recovery.^[14]^ Taken together with the results showing correlations between NLR and ENoG, the present study showed that a more severe axonal loss in the facial nerve was associated with a higher NLR and greater severity of facial weakness, as determined by a higher initial H-B grade. Because Wallerian degeneration requires time to become electrophysiologically detectable, ENoG is generally more informative several days after symptom onset.^[26–28]^ In contrast, NLR can be obtained at the initial clinical encounter using a routine blood test. In this context, NLR should not be interpreted as a replacement for ENoG, but rather as an early adjunctive marker that may help identify patients who require closer monitoring and timely electrophysiologic evaluation.

An NLR cutoff of < 1.92, determined by ROC analysis, predicted good ENoG results (≥10%) with a sensitivity of 66.7% and a specificity of 72.0%. In the CART model, which considered multiple clinical and laboratory variables, initial H-B grade and NLR were selected as the optimal variables for predicting good ENoG results. The first partitioning predictor was initial H-B grade II, and NLR < 1.61 was identified as the second partitioning predictor among patients with initial H-B grades greater than II. With the combination of initial H-B grade and NLR determined by the CART model, good ENoG results could be predicted with a sensitivity of 87.5%, a specificity of 80.0%, and an accuracy of 85.6%. Therefore, measuring NLR and evaluating initial facial weakness by H-B grade at the onset of Bell’s palsy may help predict subsequent ENoG-defined axonal loss before ENoG is performed 10 to 14 days after symptom onset. Given that NLR is an inexpensive and readily available biomarker, the combination of NLR and initial H-B grade may serve as an adjunctive marker for early risk stratification. However, these parameters should complement, rather than replace, electrophysiological evaluation.

The present study also found that older age and the presence of diabetes mellitus or hypertension were not related to the ENoG test results, which showed the degree of axonal loss 2 weeks after symptom onset. Although these clinical factors are associated with unfavorable outcomes in patients with Bell’s palsy,^[14]^ they may be more closely related to subsequent regenerative capacity than to the initial degree of axonal degeneration. In other words, ENoG primarily reflects early axonal loss, whereas age, diabetes mellitus, and hypertension may have a greater influence on long-term recovery through impaired axonal regeneration. Diabetes mellitus is associated with poor axon regeneration, which is attributed to microangiopathy and impairments in the reparative activities of Schwann cells.^[29]^ Aging is also related to a decline in axonal regeneration due to changes in neuronal, axonal, and Schwann cell responses.^[29,30]^

This study has several limitations. First, this study had a retrospective design, and the number of patients was relatively small. Because this was a single-center retrospective study, selection bias and residual confounding cannot be excluded. Therefore, larger prospective studies with long-term follow-up and external validation are needed to confirm whether NLR is directly associated with ENoG results in patients with early-stage Bell’s palsy. Second, although this study showed that ENoG results correlated with NLR and initial H-B grade in early Bell’s palsy, it did not evaluate the relationship between serial changes in NLR, ENoG-defined axonal loss, and long-term functional recovery. Therefore, longitudinal validation is required to determine whether NLR improves prognostic prediction beyond established clinical and electrophysiological parameters. Third, although multivariable analysis was performed, the number of patients with poor ENoG results was limited, which may affect the stability of the regression model. Fourth, although we used the CART method to determine the optimal combination of variables related to ENoG results, the CART model was not externally validated, and the effects of NLR within each initial H-B grade subgroup could not be fully evaluated because of the relatively small sample size.

## 5. Conclusion

In conclusion, this retrospective study showed that the results of ENoG performed 10 to 14 days after the onset of disease could objectively show the degree of axonal damage to the facial nerves and that ENoG results correlated with NLR in patients with early Bell’s palsy. In addition, good ENoG results were associated with a lower initial H-B grade. By measuring NLR at the onset of Bell’s palsy and initial facial weakness evaluated by H-B grade, it may be possible to predict subsequent axonal loss calculated by ENoG, which is performed 10 to 14 days after symptom onset.

## Author contributions

**Conceptualization:** Seongmin Choi.

**Data curation:** Seongmin Choi.

**Formal analysis:** Seongmin Choi.

**Methodology:** Myung Chul Yoo.

**Writing – original draft:** Seongmin Choi.

**Writing – review & editing:** Myung Chul Yoo.
